# Acute-Phase CD8 T Cell Responses That Select for Escape Variants Are Needed to Control Live Attenuated Simian Immunodeficiency Virus

**DOI:** 10.1128/JVI.00909-13

**Published:** 2013-08

**Authors:** Max Harris, Charles M. Burns, Ericka A. Becker, Andrew T. Braasch, Emma Gostick, Randall C. Johnson, Karl W. Broman, David A. Price, Thomas C. Friedrich, Shelby L. O'Connor

**Affiliations:** Department of Pathology and Laboratory Medicine, University of Wisconsin, Madison, Wisconsin, USAa; Wisconsin National Primate Research Center, University of Wisconsin, Madison, Wisconsin, USAb; Cardiff University School of Medicine, Heath Park, Cardiff, United Kingdomc; BSP CCR Genetics Core, SAIC-Frederick, Frederick National Laboratory, Frederick, Maryland, USAd; Chaire de Bioinformatique, Conservatoire National des Arts et Mètiers, Paris, Francee; Department of Biostatistics and Medical Informatics, University of Wisconsin, Madison, Wisconsin, USAf; Department of Pathobiological Sciences, University of Wisconsin, Madison, Wisconsin, USAg

## Abstract

The overall CD8 T cell response to human/simian immunodeficiency virus (HIV/SIV) targets a collection of discrete epitope specificities. Some of these epitope-specific CD8 T cells emerge in the weeks and months following infection and rapidly select for sequence variants, whereas other CD8 T cell responses develop during the chronic infection phase and rarely select for sequence variants. In this study, we tested the hypothesis that acute-phase CD8 T cell responses that do not rapidly select for escape variants are unable to control viral replication *in vivo* as well as those that do rapidly select for escape variants. We created a derivative of live attenuated SIV (SIVmac239Δnef) in which we ablated five epitopes that elicit early CD8 T cell responses and rapidly accumulate sequence variants in SIVmac239-infected Mauritian cynomolgus macaques (MCMs) that are homozygous for the M3 major histocompatibility complex (MHC) haplotype. This live attenuated SIV variant was called m3KOΔnef. Viremia was significantly higher in M3 homozygous MCMs infected with m3KOΔnef than in either MHC-mismatched MCMs infected with m3KOΔnef or MCMs infected with SIVmac239Δnef. Three CD8 T cell responses, including two that do not rapidly select for escape variants, predominated during early m3KOΔnef infection in the M3 homozygous MCMs, but these animals were unable to control viral replication. These results provide evidence that acute-phase CD8 T cell responses that have the potential to rapidly select for escape variants in the early phase of infection are needed to establish viral control *in vivo*.

## INTRODUCTION

The CD8 T cell response to human/simian immunodeficiency virus (HIV/SIV) is an aggregate of multiple, discrete epitope-specific responses. Patterns of T cell immunodominance are often used to categorize the antiviral efficacy of these individual CD8 T cell responses. At any given time, the most immunodominant CD8 T cell response is the highest frequency epitope-specific CD8 T cell response, while other so-called “subdominant” CD8 T cell responses are present at lower individual frequencies (1). The earliest immunodominant CD8 T cell responses contribute to the establishment of the viral load set point and define the likelihood of long-term viral control within an individual (2, 3), while the subdominant CD8 T cell responses contribute to effective control of chronic HIV/SIV replication (4, 5). Alternatively, the antiviral efficacy of CD8 T cells can be defined by whether they select for immune escape variants rapidly or slowly. Epitopes that do not rapidly accumulate variants have become attractive as vaccine immunogens because they are common in circulating strains of HIV (6). Nonetheless, it remains unclear to what extent CD8 T cell responses specific for epitopes that rapidly accumulate variants and those specific for epitopes that remain invariant can control early viral replication. Knowledge of which populations of CD8 T cells can best suppress viral replication is needed to design a T cell-based vaccine.

It has been difficult to define whether acute-phase CD8 T cell responses targeting invariant epitopes can establish control of HIV replication. The development of individual epitope-specific CD8 T cell responses is dependent on both the total repertoire of HLA/major histocompatibility complex (MHC) class I alleles in the host and the sequence of the infecting virus. A “pre-escaped” HIV can be transmitted between individuals who share a single HLA class I allele, but acute-phase CD8 T cell responses that develop in the newly infected individual can still select for escape variants in epitopes that did not accumulate variants in the donor (7–9). Although the efficacy of acute-phase CD8 T cell responses targeting invariant epitopes could be measured when a pre-escaped HIV is transmitted between fully HLA-identical people, this event is exceptionally rare. Thus, it is nearly impossible to evaluate the *in vivo* antiviral efficacy of acute-phase CD8 T cell responses that do not rapidly select for variants in HIV-positive individuals.

Nonhuman primate studies with engineered SIV strains containing point mutations in T cell epitopes can be used to model the transmission of pre-escaped HIV. These studies typically focus on responses restricted by a single MHC class I allele, but CD8 T cell responses targeting epitopes restricted by alternate MHC class I alleles can develop and rapidly select for escape variants. Not surprisingly, control of pre-escaped SIV replication has been inconsistent and unpredictable in these studies (10–12) and the antiviral efficacy of acute-phase CD8 T cell responses targeting invariant epitopes could not be evaluated. Fully MHC class I-matched Mauritian cynomolgus macaques (MCMs) infected with SIV can overcome these limitations by mounting consistent CD8 T cell responses that select for sequence variants in the same epitopes (13, 14). MCMs that are homozygous for the M3 MHC class I haplotype are present at a frequency of ∼4% (15). There are now 14 known epitopes restricted by MHC class I molecules encoded by the M3 haplotype (13, 16–18), of which 5 consistently accumulate sequence variants within the first 12 weeks after SIVmac239 infection in M3 homozygous MCMs. With such exquisite knowledge of host MHC genetics and epitope-specific CD8 T cell immunity, this model can be customized to evaluate the antiviral efficacy of CD8 T cell responses that do not rapidly select for escape variants.

We took advantage of the unique replication kinetics of the live attenuated virus SIVmac239Δnef to understand the contribution of CD8 T cell responses that do not rapidly select for escape variants on elite viral control. In Indian rhesus macaques and Mauritian cynomolgus macaques infected with SIVmac239Δnef, an acute-phase peak of virus replication is subsequently controlled to levels that are not detectable with plasma viral load assays (19, 20). Viral load decline is coincident with the emergence of CD8 T cell responses (21, 22) and can be used as an indicator of T cell function.

We created an SIVmac239Δnef derivative, termed m3KOΔnef, in which we ablated four of the five epitopes that accumulate variants within 12 weeks of SIVmac239 infection. The fifth epitope is in an alternate reading frame and was undefined when we created m3KOΔnef, so it remained intact. We eliminated one additional well-characterized epitope in Pol that frequently accumulates variants by 14 weeks after infection (18). M3 homozygous MCMs in the acute phase of infection with m3KOΔnef are therefore capable of developing acute-phase CD8 T cell responses that target less variable epitopes, making this the first model available to assess the functional potency of these CD8 T cell responses to control early SIV replication. We found that M3 homozygous MCMs could not control m3KOΔnef as well as MCMs that did not have any of the MHC class I alleles present in the M3 MHC haplotype. These results provide compelling evidence that CD8 T cell responses that do not rapidly select for escape variants have limited potency *in vivo* and argue that caution should be used when designing vaccines to elicit T cell responses that target conserved epitopes.

## MATERIALS AND METHODS

### Creation of virus stocks.

The following clonal virus stocks were created for this study: SIVmac239Δnef, barcoded SIVmac239Δnef (BCVΔnef), and m3KOΔnef. For SIVmac239Δnef, we obtained the necessary plasmids containing the 5′ and 3′ viral genomes (p239SpSp5′ and pSIVmac239Δnef deletion mutant) from Ronald Desrosiers through the AIDS Research and Reference Reagent Program, Division of AIDS, NIAID, NIH. The plasmid containing the 5′ SIV genome with 10 synonymous “barcoding” mutations in Gag was used previously (12). For m3KOΔnef, we first identified consensus variants that accumulated in virus populations replicating in M3^+^ MCMs during chronic SIVmac239 infection (15; data not shown). Twenty-three substitutions were then incorporated into SIVmac239Δnef by custom gene synthesis (GenScript, Piscataway, NJ) to create the 5′ and 3′ plasmids necessary to construct m3KOΔnef. These plasmids were designed to include the SphI restriction site required for coligation, and they contained long terminal repeat sequences matching those in SIVmac239.

All virus stocks were created as described previously (23, 24). The plasmids containing the 5′ and 3′ halves of the corresponding genomes were digested with SphI, treated with shrimp alkaline phosphatase, precipitated, and then ligated together. Vero cells were transfected with the ligated products and then cocultured with CEMx174 cells for 48 h. Infected CEMx174 cells were grown for about 1 week to produce high-titer viruses. The p27 content of each virus stock was determined by enzyme-linked immunosorbent assay (ELISA; ZeptoMetrix, Buffalo, NY) according to the manufacturer's protocol. The infectious titer of each virus stock was determined by limiting-dilution inoculation of CEMx174 cells. Briefly, cells were inoculated in quadruplicate with serial 4-fold dilutions of virus stock, beginning at a 1:100 dilution. Two weeks later, individual cultures were scored as positive or negative for SIV infection by qualitative p27 antigen capture ELISA (ZeptoMetrix). The infectious titer was then estimated by using the proportionate-distance method of Reed and Muench (25) and expressed in terms of 50% tissue culture infectious doses (TCID_50_) per milliliter of virus stock.

### *In vitro* coculture competition assays.

All virus stocks were normalized for a p27 content of 50 ng/ml. Viruses were mixed in the following combinations: 2.5 ng m3KOΔnef or SIVmac239Δnef to 2.5 ng BCVΔnef (1:1), 0.5 ng m3KOΔnef or SIVmac239Δnef to 4.5 ng BCVΔnef (1:9), and 4.5 ng m3KOΔnef or SIVmac239Δnef to 0.5 ng BCVΔnef (9:1). Each virus mixture was incubated with 10^6^ CEMx174 cells at 37°C for 4 h. After washing, 5 × 10^5^ cells were plated and grown for 1 week. Supernatant was collected at days 3, 5, and 7.

The copy number of each virus was measured in the inoculum and in each supernatant. Viral RNA was isolated with the M48 Biorobot apparatus (Qiagen, Valencia, CA). Quantification was conducted with the SuperScript III Platinum One-Step quantitative PCR kit (Invitrogen, Carlsbad, CA) as previously described (12). The SIVmac239Δnef and m3KOΔnef viruses were quantified with primers and probes targeting an 84-bp region of *gag* (12, 26). BCVΔnef was quantified in a separate reaction with a distinct set of primers and probes (12). The appropriate wild-type or barcoded transcript was used for each standard curve. All comparative reactions were run on the same plate with a Roche LightCycler 480 under the following conditions: cDNA synthesis at 50°C for 30 min, an initial denaturation step at 95°C for 2 min, and 45 amplification cycles of 95°C for 10 s and 69°C for 30 s.

The ratio of m3KOΔnef or SIVmac239Δnef to BCVΔnef was determined for each sample and then normalized to the ratio that was present in the inoculum. Replicative differences between viruses were assessed at each time point with unpaired *t* tests (GraphPad Prism, La Jolla, CA). All data were plotted on a log_2_ scale.

### Animal care and use.

MCMs were purchased from Charles River Laboratories and cared for by the Wisconsin National Primate Research Center according to protocols approved by the University of Wisconsin Graduate School Animal Care and Use Committee. Animals CY0348, CY0379, CY0381, CY0382, CY0383, CY0384, and CY0385 were infected intravenously with 10 ng of p27 m3KOΔnef. Animals CY0332, CY0333, CY0334, CY0335, CY0336, and CY0337 were infected intrarectally with 7,000 TCID_50_ of SIVmac239 as part of another study (13, 16). Animals CY0114, CY0157, CY0160, CY0205, CY0206, CY0213, CY0338, CY0345, and CY0386 were infected intravenously with 10 ng of p27 SIVmac239Δnef (19).

### Plasma viral loads analysis.

Plasma SIV loads were determined essentially as previously described (15, 26). Viral RNA was isolated from plasma and then reverse transcribed and amplified with the SuperScript III Platinum one-step quantitative reverse transcription (RT)-PCR system (Invitrogen). Samples were quantified on a Roche LightCycler 2.0 and compared to an internal standard curve on each run. The substitutions present in m3KOΔnef did not lie within the primer or probe target sequence.

Differences in the log_10_ viral load set point were modeled by linear mixed-effects regression with random effects for each animal and fixed effects for each group. The mean log_10_ viral load set point for a group was estimated by viral load measurements between weeks 14 and 20 after infection. The maximum log_10_ viral load before 5 weeks was defined as the acute-phase peak viral load, and differences were modeled by linear regression. Statistics and significance measures were calculated in R 2.15.2 with the lmer and gmodels packages.

### Genome-wide sequencing of SIV and data analysis.

Genome-wide sequencing of replicating SIV was performed essentially as previously described (13, 27). Viral RNA was isolated from plasma with the QIAamp MinElute Virus Spin kit (Qiagen). The Superscript III One-Step RT-PCR system with Platinum *Taq* High Fidelity (Invitrogen) was used to reverse transcribe the viral RNA in four overlapping amplicons spanning the entire SIV genome. PCR products were purified with the Qiagen MinElute Gel Extraction kit (Qiagen) and quantified with the Quant-IT dsDNA HS Assay kit (Invitrogen).

For pyrosequencing, libraries were prepared with the Nextera DNA Sample Prep kit (Roche Titanium compatible; Epicentre, Madison, WI) and 10-bp multiplex identifier tags were added. Tagged products were purified twice with Agencourt AMPure XP beads (Beckman Coulter Genomics, Danvers, MA) and then quantified with the Quant-IT dsDNA HS Assay kit (Invitrogen) and the Agilent High Sensitivity DNA kit (Agilent Technologies, Santa Clara, CA). Pyrosequencing was performed with a Roche/454 GS Junior instrument with Titanium shotgun chemistry, according to the manufacturer's protocols (454 Life Sciences, Branford, CT).

Nucleotide variation relative to SIVmac239 was determined at each position with Galaxy, an online tool used to analyze next-generation sequence data (13, 28–30). FASTQ reads were trimmed, and low-quality uncertain sequences were masked with an “N.” Sequence reads were mapped to an SIVmac239 reference (accession no. M33262) with the LASTZ algorithm. The SAM format was converted to a BAM format, and a pileup was created. The total A, C, T, G, and N numbers at each site were calculated. Sites that were greater than 1% variant from the reference were assessed as synonymous or nonsynonymous with the SNPeffect tool (31). Variants spanning nucleotide positions 1300 to 10200 with >1% variation and greater than three times the percentage of uncertain or indel sequences were plotted with GraphPad Prism.

Amino acid variation relative to SIVmac239 was determined as described previously (13, 16). FASTQ reads were trimmed, and low-quality sequences were masked with an “N.” FASTQ reads were converted to FASTA reads and translated in all six reading frames. BLAT (BLAST-like alignment tool) was used to align sequence reads with SIVmac239 proteins at a minimum of 50% identity. Custom scripts were used to extract the region of interest, remove reads with ambiguous sequences, and measure the frequency of each variant identified.

### IFN-γ ELISPOT assays.

Gamma interferon (IFN-γ) enzyme-linked immunospot (ELISPOT) assays were performed as described previously (15, 18). First, peripheral blood mononuclear cells (PBMCs) were isolated from EDTA-anticoagulated blood by Ficoll-Paque Plus (GE Healthcare, Piscataway, NJ) density gradient centrifugation. A precoated monkey IFN-γ ELISPOTplus plate (Mabtech, Mariemont, OH) was blocked, and individual peptides were added to each well at a final concentration of 1 μM. Each peptide was tested in duplicate, and concanavalin A (10 μM) was used as a positive control. Assays were performed according to the manufacturer's protocol, and wells were imaged with an AID ELISPOT reader. The average number of spots per peptide was calculated, and we subtracted the average number of spots formed with no peptide. Data were extrapolated to 10^6^ PBMCs to calculate the number of spot-forming cells (SFCs) per 10^6^ PBMCs.

### Generation of peptide-specific T cell lines.

T cell lines were grown as described previously (17, 18). First, 5 × 10^6^ PBMCs were isolated from CY0381 and incubated with the immunogenic peptide (either ARF1_29-43_VY15 or ARF1_30-40_QL11) for 1 week in medium supplemented with 5 × 10^5^ U/ml of interleukin-2 (Prometheus Laboratories, San Diego, CA). Restimulation was conducted every 1 to 2 weeks with autologous irradiated B-lymphoblastoid cells pulsed with the relevant peptide.

### Intracellular cytokine staining.

Activation of peptide-specific T cells was measured via the production of IFN-γ and tumor necrosis factor alpha (TNF-α) as described previously (18). Briefly, peptide-pulsed autologous B-lymphoblastoid cells were used to stimulate cultured T cells at a 1:1 ratio for 4 h in the presence of brefeldin A (10 μg/ml; Sigma-Aldrich). Single MHC allele transferent cell lines, pulsed with peptide and washed extensively, were used similarly to determine MHC restriction. Cells were then stained with anti-CD8–PacBlue (BD Biosciences, San Jose, CA) and anti-CD3–AF700 (BD Biosciences) antibodies, fixed with 2% paraformaldehyde, and left at 4°C overnight. On the following morning, the cells were permeabilized in 1% saponin buffer, stained with anti-IFN-γ–fluorescein isothiocyanate (FITC) (BD Biosciences) and anti-TNF-α–phycoerythrin (PE) (BD Biosciences) antibodies, washed in 1% saponin buffer, and fixed in 2% paraformaldehyde. Flow cytometry was performed with an LSRII instrument (BD Biosciences) and data were analyzed with FlowJo V.9.4.10 software (TreeStar Inc., Ashland, OR).

### Tetramer staining.

Fluorochrome-conjugated MHC/peptide tetramers were produced as described previously (32). The tetramers used in this study were Mafa-A1*063/ARF1_30-40_QL11-PE, Mafa-A1*063/Env_338-346_RF9-PE, Mafa-B*075/Tat_42-49_QA8-PE, and Mafa-B*075/Rev_59-68_SP10-PE. Thawed lymph node cells or PBMCs were stained with tetramer at 37°C for 20 min, washed, and then stained with anti-CD3–AF700 (BD Biosciences), anti-CD8–PacBlue (BD Biosciences), and anti-CD69–Texas Red–PE (Beckman Coulter) antibodies. Flow cytometry was performed with an LSRII instrument (BD Biosciences), and data were analyzed with FlowJo V.9.4.10 software (TreeStar Inc.). Tetramer-binding CD8 T cell frequencies were compared between groups with paired or unpaired two-tailed *t* tests (GraphPad Prism) after log transformation of the data.

### Measurement of immune activation.

Immune activation assays were based on previous reports (33). Whole blood or cells isolated from bronchoalveolar lavage (BAL) fluid were first incubated with surface stains. Blood samples were then treated with FACSlyse, and BAL fluid samples were treated with 2% paraformaldehyde. Cells were then permeabilized in 0.1% saponin buffer, stained with antibodies specific for intracellular markers, washed, and analyzed by flow cytometry. The antibodies used were anti-CD38–FITC (Stem Cell Technologies, Inc.), anti-CD4–PeCy7 (BD Biosciences), anti-CD3–PacBlue (BD Biosciences), anti-Ki-67–AF647 (BD Biosciences), anti-CD20–AF700 (BioLegend), anti-CD8–APCCy7 (BioLegend, San Diego, CA), and anti-CD69–Texas Red–PE (Beckman Coulter, Indianapolis, IN).

## RESULTS

### Creation of an SIVmac239Δnef variant that is pre-escaped in CD8 T cell epitopes.

**Fig 1:**
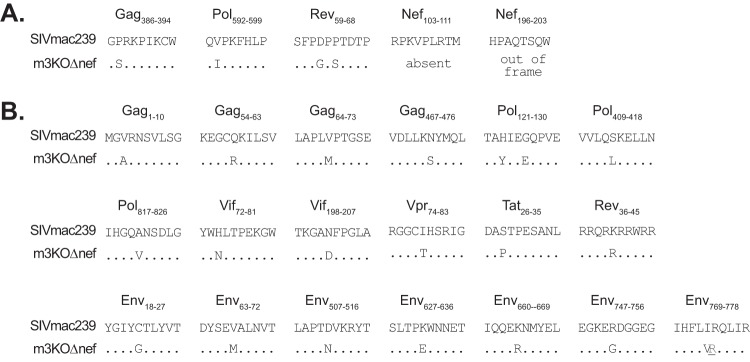
Amino acid sequence differences between SIVmac239 and m3KOΔnef. Twenty-three nucleotide mutations that affected 24 amino acid sites were engineered into SIVmac239Δnef to create m3KOΔnef. The SIV proteins and the amino acid positions affected are shown. Dots represent identity, and capital letters represent amino acid differences. The underlined letter in light gray indicates a nucleotide change that was synonymous with respect to that specific protein. (A) Substitutions engineered into epitopes restricted by MHC class I molecules encoded by the M3 MHC haplotype are shown. (B) Additional substitutions outside known epitopes are shown.

We previously infected four M3 homozygous MCMs with SIVmac239 and found that three of them established high viral load set points (viral loads, >10^6^ copies/ml) (15, 27). Since few CD8 T cell epitopes restricted by molecules encoded by the M3 MHC haplotype had been identified when we began this study, we reasoned that high-frequency variants shared between virus populations isolated from these three M3 homozygous MCM progressors would have adequate replicative fitness and contain effective escape mutations in commonly recognized CD8 T cell epitopes. We identified 23 shared, high-frequency nucleotide variants that were nonsynonymous at 24 amino acid sites in the nine main SIV reading frames and introduced these variant nucleotides into SIVmac239Δnef (Fig. 1). We termed the resulting virus m3KOΔnef to denote its origin as an SIVmac239Δnef derivative specifically deficient in putative CD8 T cell epitopes restricted by alleles present in the M3 MHC haplotype.

Fourteen CD8 T cell epitopes from SIVmac239 that are restricted by MHC class I molecules encoded by the M3 MHC haplotype were subsequently identified (16, 17), including one epitope defined in this study that is derived from a cryptic open reading frame (see Fig. S1 in the supplemental material). Five of these epitopes (Gag_386-394_GW9, Pol_592-599_QP8, Rev_59-68_SP10, Nef_103-111_RM9, and Nef_196-203_HW8) were disrupted with five of the nucleotide substitutions and the deletion in *nef* (Fig. 1A). All five of these epitopes accumulate variants by 14 weeks after SIVmac239 infection in M3 homozygous MCMs (13, 16, 18). Accordingly, we could determine whether MCMs could control the replication of attenuated SIV in the absence of CD8 T cell responses targeting five epitopes that escape during the acute phase of infection.

We reasoned that there were nine epitopes restricted by MHC class I molecules encoded by the M3 haplotype that were intact in m3KOΔnef. Of these nine epitopes, the cryptic epitope (ARF1_30-40_QL11) was the only one that accumulated variants by 12 weeks after SIVmac239 infection. This left eight epitopes that elicit CD8 T cell responses that do not commonly select for variants during the acute phase of SIVmac239 infection. Accordingly, we could determine whether CD8 T cell responses targeting the remaining intact epitopes could effectively control replication of m3KOΔnef. The additional 19 nucleotide substitutions that were introduced to create m3KOΔnef were typically found in virus populations replicating in SIVmac239-infected M3 homozygous MCMs, but the role of these other variants is unknown.

We tested the fitness of m3KOΔnef by using *in vitro* coculture competition assays with a barcoded SIVmac239Δnef (BCVΔnef) (12). Our data indicated that the mutations we incorporated into SIVmac239Δnef to create m3KOΔnef did not decrease viral fitness *in vitro* (see Fig. S2 in the supplemental material). In fact, m3KOΔnef appeared to replicate slightly more efficiently than SIVmac239Δnef *in vitro*.

### M3 homozygous MCMs do not control replication of m3KOΔnef.

We infected four M3 homozygous MCMs and three MHC-mismatched MCMs with m3KOΔnef (Table 1). M3 homozygous animals only express MHC alleles present in the M3 MHC haplotype. None of the major MHC class I alleles present in the MHC-mismatched MCMs are present in the M3 haplotype (34). Most of the animals in the MHC-diverse group expressed MHC alleles that are also present in the M3 MHC haplotype, but none were M3 homozygotes. Although a group of M3 homozygous MCMs infected with SIVmac239Δnef would have been valuable for direct comparison, we were unable to obtain this additional group for our study.

**Table 1:** Animals used in this study

Animal no.	MHC genotype	Infecting virus
CY0379	M3/M3	m3KOΔnef
CY0381	M3/M3	m3KOΔnef
CY0384	M3/M3	m3KOΔnef
CY0385	M3/M3	m3KOΔnef
CY0348	M4/M4	m3KOΔnef
CY0382	M4/M5	m3KOΔnef
CY0383	M4/M7	m3KOΔnef
CY0332	M3/M3	SIVmac239
CY0333	M3/M3	SIVmac239
CY0334	M3/M3	SIVmac239
CY0335	M3/M3	SIVmac239
CY0336	M3/M3	SIVmac239
CY0337	M3/M3	SIVmac239
CY0114	M1/M3	SIVmac239Δnef
CY0157	M1/M1	SIVmac239Δnef
CY0160	M1/M2	SIVmac239Δnef
CY0205	M1/M3	SIVmac239Δnef
CY0206	M1/M3	SIVmac239Δnef
CY0213	M1/M3	SIVmac239Δnef
CY0338	M1/M2	SIVmac239Δnef
CY0345	M1/M3	SIVmac239Δnef
CY0386	M1/M3	SIVmac239Δnef

**Fig 2:**
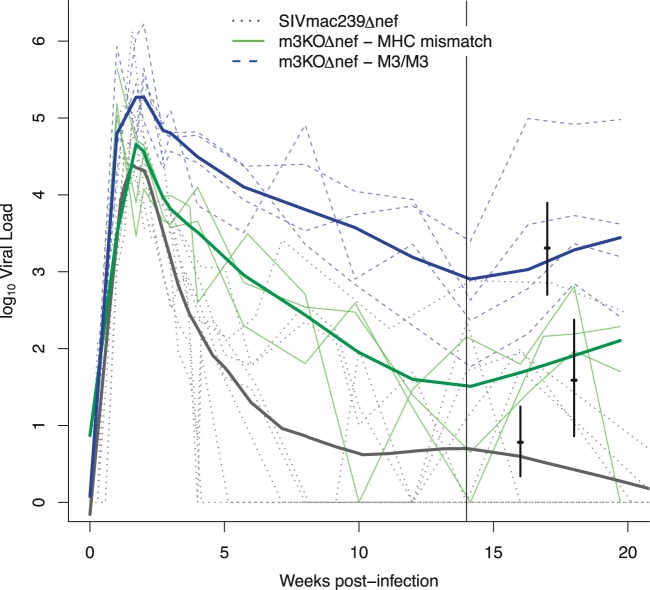
Virus replication is not controlled in M3 homozygous MCMs infected with m3KOΔnef. Viral loads were measured for 20 weeks after infection of the following groups: M3 homozygous MCMs infected with m3KOΔnef (blue), MHC-mismatched MCMs infected with m3KOΔnef (green), and MHC-diverse MCMs infected with SIVmac239Δnef (black). All MHC genotypes are shown in Table 1. Log_10_ viral load trajectories are shown for each individual (thin lines) and each group (smoothed, heavy lines). Viral load set point estimates and 95% confidence intervals for each group are represented by short vertical bars (see Table 2 for statistics). The tall vertical black line is a reference point at 14 weeks after infection.

We observed a small increase in the peak plasma viremia of m3KOΔnef in M3 homozygous MCMs compared to the peak viremia of SIVmac239Δnef in MHC-diverse MCMs (*P* = 0.03, Fig. 2 and Table 2). There were no other significant differences between the peak viral loads. The similar peak viremias observed in the MHC-mismatched MCMs infected with m3KOΔnef and the MHC-diverse MCMs infected with SIVmac239Δnef suggest that the mutations we incorporated into SIVmac239Δnef to create m3KOΔnef did not reduce viral fitness *in vivo*.

**Table 2:** Peak viral loads in groups of animals infected with different strains of attenuated SIV

Group (no. of animals)	Peak viral load (log_10_)	95% CI^*a*^ (log_10_)	
		Lower	Upper
M3 homozygous MCMs infected with m3KOΔnef (4)	5.78^*b*^	5.23	6.32
MHC-mismatched MCMs infected with m3KOΔnef (3)	5.53	4.98	6.08
MHC-diverse MCMs infected with SIVmac239Δnef (8)	5.02	4.63	5.41

a CI, confidence interval.

b M3 homozygous MCMs infected with m3KOΔnef versus MHC-diverse MCMs infected with SIVmac239Δnef, *P* = 0.03.

The mean viral load set point established between 14 and 20 weeks after infection was significantly higher in the M3 homozygous MCMs than in the MHC-mismatched MCMs infected with m3KOΔnef (*P* < 0.001, Fig. 2 and Table 3), demonstrating that M3 homozygous MCMs are unable to control m3KOΔnef replication. Although the mean viral load set point was higher in the MHC-mismatched MCMs infected with m3KOΔnef than in historical control MCMs infected with SIVmac239Δnef (*P* = 0.058, Fig. 2 and Table 3; David O'Connor and Justin Greene, personal communication), these were not significantly different. Thus, suppression of m3KOΔnef replication can occur in animals without M3 MHC class I alleles.

**Table 3:** Mean viral load set points in groups of animals infected with different strains of attenuated SIV

Group (no. of animals)	Mean viral load set point (log_10_)	95% CI^*a*^ (log_10_)	
		Lower	Upper
M3 homozygous MCMs infected with m3KOΔnef (4)	3.31^*b*^^,^^*c*^	2.70	3.90
MHC-mismatched MCMs infected with m3KOΔnef (3)	1.59^*d*^	0.86	2.37
MHC-diverse MCMs infected with SIVmac239Δnef (8)	0.78	0.34	1.25

a CI, confidence interval.

b M3 homozygous MCMs infected with m3KOΔnef versus MHC-diverse MCMs infected with SIVmac239Δnef, *P* < 0.001.

c M3 homozygous MCMs infected with m3KOΔnef versus MHC-mismatched MCMs infected with m3KOΔnef, *P* < 0.001.

d MHC-mismatched MCMs infected with m3KOΔnef versus MHC-diverse MCMs infected with SIVmac239Δnef, *P* = 0.058.

**Fig 3:**
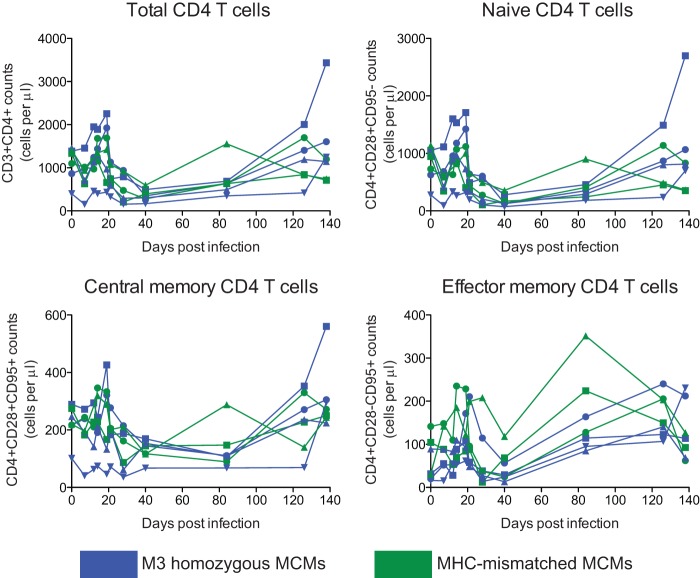
CD4 T cell counts in MCMs infected with m3KOΔnef. PBMCs were stained during the first 20 weeks after infection with m3KOΔnef with anti-CD3, -CD4, -CD8, -CD95, and -CD28 antibodies. Samples were analyzed by flow cytometry. Absolute counts are shown for the whole CD4 T cell compartment and various CD4 T cell subsets, as indicated.

We measured the depletion of peripheral CD4 T cells after infection with m3KOΔnef in all seven animals. In contrast to the preservation of peripheral CD4 T cells in macaques infected with SIVmac239Δnef (35, 36), we found that peripheral CD4 T cell levels declined during the acute phase of infection in all of the animals, independently of the MHC genotype, and then rebounded (Fig. 3).

We also observed greater frequencies of CD4 and CD8 T cells in the BAL fluid and blood samples that were both CD38^+^ and Ki67^+^ during the acute m3KOΔnef infection phase than in those animals infected with SIVmac239Δnef (see Table S1 and Fig. S3 in the supplemental material). This was most apparent in cells isolated from BAL fluid at 14 days after infection. These data provide evidence that there was greater immune activation in the animals infected with m3KOΔnef than in animals infected with SIVmac239Δnef. This was not surprising, as high levels of immune activation have been observed during the acute phase of viral infection (37, 38). Even though our data suggest that m3KOΔnef was potentially more pathogenic than SIVmac239Δnef, replication of m3KOΔnef was more susceptible to host immune responses in animals without M3 MHC alleles than in those animals with M3 MHC alleles.

### Detection of CD8 T cell responses in the blood and lymph nodes of MCMs infected with m3KOΔnef.

**Fig 4:**
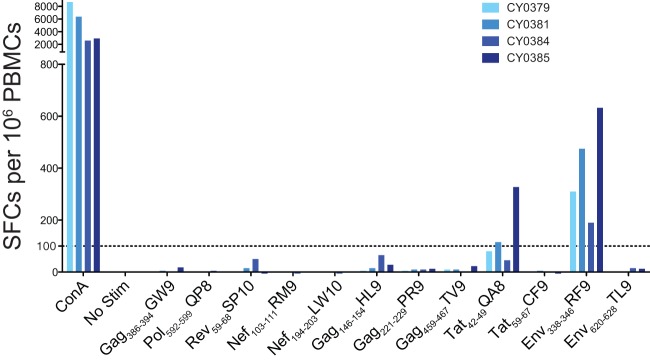
CD8 T cell responses targeting disrupted epitopes are absent during the acute phase of infection of M3 homozygous MCMs with m3KOΔnef. IFN-γ ELISPOT assays were performed with PBMCs from M3 homozygous MCMs at 3 weeks after infection with m3KOΔnef to measure T cell responses targeting 12 SIV-derived peptide epitopes that are restricted by M3 MHC class I molecules. Samples were plated in duplicate, and the average numbers of SFCs per 10^6^ PBMCs are shown. The Nef_194-203_LW10 peptide contains the Nef_196-203_HW8 epitope. ConA, concanavalin A; Stim, stimulation.

**Fig 5:**
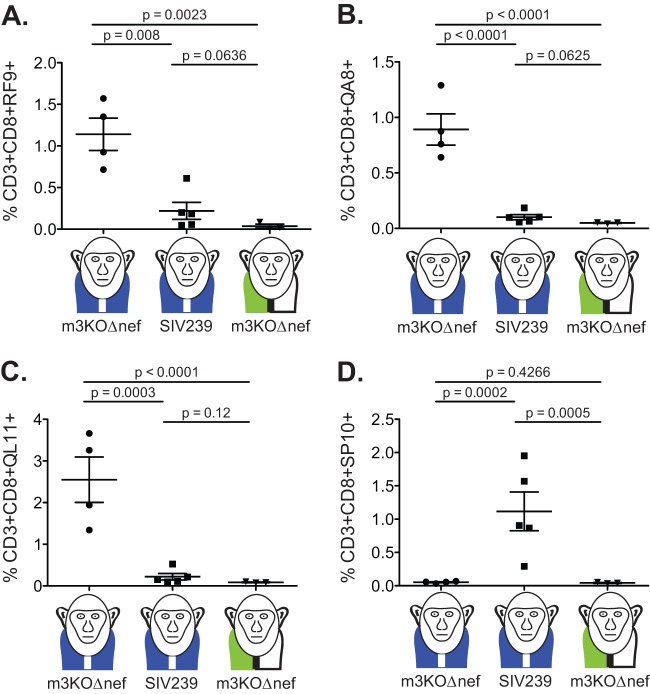
CD8 T cells detected in M3 homozygous MCMs during the acute m3KOΔnef infection phase target different epitopes compared to the acute phase of SIVmac239 infection. MHC/peptide tetramers presenting four SIVmac239-derived epitopes were used to quantify the percentage of antigen-specific CD3^+^ CD8^+^ T cells in lymph nodes at 3 weeks after infection with the indicated viruses. The specificities examined were Env_338-346_RF9 (A), Tat_42-49_QA8 (B), ARF1_30-40_QL11 (C), and Rev_59-68_SP10 (D). Each point represents a different animal. The monkeys with blue vests represent M3 homozygous MCMs; the monkeys with green-and-white vests represent MHC-mismatched MCMs. Unpaired *t* tests of the log-transformed data were performed, and the *P* values are shown.

We next examined which epitopes were targeted by acute-phase CD8 T cell responses in M3 homozygous MCMs infected with m3KOΔnef in the peripheral blood and lymph nodes. As expected, CD8 T cell responses targeting the five epitopes that were ablated in m3KOΔnef were undetectable at 3 weeks after m3KOΔnef infection, as measured by IFN-γ ELISPOT assays with PBMCs and tetramer staining of cells isolated from lymph nodes (Fig. 4 and 5D). These same animals, however, had acute-phase CD8 T cell responses targeting two alternate CD8 T cell epitopes (Env_338-346_RF9 and Tat_42-49_QA8) that typically do not accumulate variants until the chronic infection phase (Fig. 4 and 5A and B) (27). We also detected CD8 T cells in lymph nodes targeting the ARF1_30-40_QL11 cryptic epitope (Fig. 5C) that was identified in this study (see Fig. S1 in the supplemental material). The detection of three acute-phase CD8 T cell responses in M3 homozygous MCMs infected with m3KOΔnef demonstrated that the remaining intact immunogenic epitopes had the potential to elicit epitope-specific CD8 T cell responses restricted by M3-encoded MHC class I molecules. Two of these responses typically do not rapidly select for escape variants during infection with SIVmac239, and the third response targets a cryptic epitope.

### Sequence variants accumulate more rapidly in the Env_338-346_RF9 and ARF1_30-40_QL11 epitopes after infection with m3KOΔnef.

**Fig 6:**
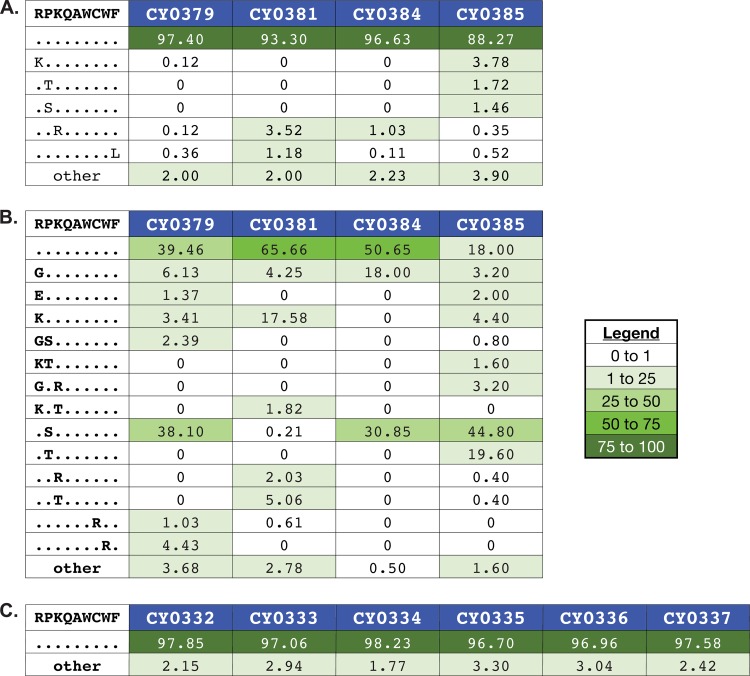
Accumulation of sequence variants in Env_338-346_RF9 is more rapid in M3 homozygous MCMs infected with m3KOΔnef than in those infected with SIVmac239. Viruses replicating in M3 homozygous MCMs at 3 weeks after m3KOΔnef infection (A), 12 weeks after m3KOΔnef infection (B), and 12 weeks after SIVmac239 infection (C) were subjected to genome-wide sequencing by Roche/454 pyrosequencing. Amino acid variants in Env_338-346_RF9 were investigated as described in Materials and Methods. The wild-type sequence and animal identification numbers are shown at the top of each panel. Masked identical bases are represented by dots, and amino acid replacements are represented by capital letters. The frequency of each epitope variant sequence is shown. A variant sequence had to be present in at least one animal at a frequency of 1% or greater to be included in the graphic. The legend indicates the color associated with a given variant frequency. A zero indicates that no reads were detected with that sequence.

Next, we asked whether the selection of viral variants was accelerated in the three epitopes (Env_338-346_RF9, Tat_42-49_QA8, and ARF1_30-40_QL11) targeted during the acute m3KOΔnef infection phase. We deep sequenced the entire SIV coding region in M3 homozygous MCMs infected with m3KOΔnef at 3 and 12 weeks after infection (see Tables S2 and S3 in the supplemental material) and compared the data to the sequences of virus populations isolated from M3 homozygous MCMs at 4 and 12 weeks after infection with SIVmac239 (13). Very few variants were detected in the Env_338-346_RF9 epitope in virus populations isolated from M3 homozygous MCMs at week 3 after infection with m3KOΔnef (Fig. 6A), but substantial variation was apparent after 12 weeks (Fig. 6B). In contrast, variants were still not detected in the Env_338-346_RF9 epitope by 12 weeks after infection with SIVmac239 (Fig. 6C). We did not detect variation within the Tat_42-49_QA8 epitope in virus populations isolated from animals infected with either m3KOΔnef or SIVmac239 by 12 weeks after infection (data not shown).

**Fig 7:**
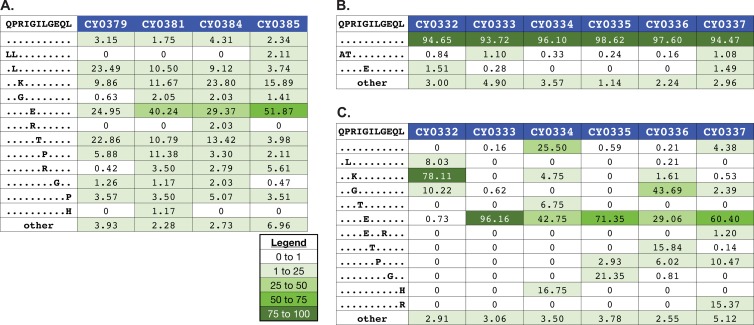
Rapid accumulation of nucleotide substitutions in the ARF1_30-40_QL11 epitope at 3 weeks after infection in M3 homozygous MCMs infected with m3KOΔnef. Virus populations were isolated and subjected to genome-wide deep sequencing. Samples came from M3 homozygous MCMs 3 weeks after infection with m3KOΔnef (A), M3 homozygous MCMs 4 weeks after infection with SIVmac239 (B), and M3 homozygous MCMs 12 weeks after infection with SIVmac239 (C). Amino acid variants in ARF1_30-40_QL11 were investigated as described in Materials and Methods. The wild-type sequence and animal identification numbers are shown at the top of each panel. Masked identical bases are represented by dots, and amino acid replacements are represented by capital letters. The frequency of a given epitope variant sequence is shown. A variant sequence had to be present in at least one animal at a frequency of 1% or greater to be included in the graphic. The legend indicates the color associated with a given variant frequency. A zero indicates that no reads were detected with that sequence.

Interestingly, we detected variation within the ARF1_30-40_QL11 epitope in M3 homozygous MCMs at 3 weeks after infection with m3KOΔnef (Fig. 7A). This time frame was on a par with the rapid accumulation of variants detected in several of the epitopes that were disrupted in m3KOΔnef (13). In M3 homozygous MCMs infected with SIVmac239, we found that this epitope did not accumulate variants 4 weeks after infection (Fig. 7B), although substantial variation was apparent after 12 weeks (Fig. 7C). Unfortunately, we did not remove the ARF1_30-40_QL11 epitope in our pre-escaped virus because we did not know it existed when we began the project. Nonetheless, variant accumulation in this epitope was more rapid in M3 homozygous MCMs infected with m3KOΔnef than in MHC-matched animals infected with SIVmac239.

In addition, we sequenced virus populations isolated from MCMs at 3 weeks after infection with SIVmac239Δnef. We did not find any nonsynonymous variants present at a frequency greater than 20% (see Fig. S4 in the supplemental material). Viral loads were too low for sequencing at later time points. With such rapid viral control, the absence of variants in these virus populations at this early time point was not surprising. We expect that variants will accumulate in SIVmac239Δnef over time, as detected previously by others (19, 39).

Notably, we did not detect reversion of the engineered mutations in virus populations isolated from any of the animals by 3 weeks after infection with m3KOΔnef, and there was minimal reversion by 12 weeks in the M3 homozygotes (see Fig. S5 and S6 in the supplemental material). The stability of the engineered substitutions further suggests that the variants introduced were not deleterious to viral replication.

## DISCUSSION

In the present study, we took advantage of MHC-identical MCMs to study CD8 T cell-mediated control of live attenuated SIV replication, an MHC-independent model of elite control. We created a derivative of SIVmac239Δnef incorporating commonly detected nucleotide substitutions identified in virus populations replicating in M3 homozygous MCMs. In m3KOΔnef, we ablated five epitopes that all accumulate variants by 14 weeks after SIVmac239 infection of M3 homozygous MCMs. Even though CD8 T cell responses targeting these five epitopes were absent, we detected reproducible CD8 T cell responses targeting three additional epitopes that remained intact in m3KOΔnef, two of which typically do not select for escape variants until the chronic infection phase in M3 homozygous MCMs infected with SIVmac239. Replication of m3KOΔnef was not controlled in M3 homozygous animals, suggesting that the set of CD8 T cell responses that predominated in M3 homozygous MCMs during the acute phase of infection with m3KOΔnef and do not tend to rapidly select for escape variants were unable to control viral replication.

The key observation in this study is that control of live attenuated SIV replication is strongly associated with acute-phase CD8 T cell responses that rapidly select for sequence variants. We found that CD8 T cell responses that do not typically select for escape variants during the first few weeks of SIV infection were insufficient to control the replication of m3KOΔnef. In contrast, m3KOΔnef replication was controlled in animals with entirely different MHC class I alleles, enabling the presentation of alternate epitopes. These results suggest that the CD8 T cell responses that typically emerge during the chronic infection phase and do not rapidly select for escape variants are collectively less potent than the acute-phase CD8 T cell responses that do rapidly select for escape variants.

An alternate explanation for these results is that acute-phase CD8 T cell responses target some epitopes that do rapidly accumulate variants and some that do not. By specifically eliminating the responses that do select for escape variants, we inadvertently reduced the effective breadth of the acute-phase CD8 T cell response, thus reducing viral control. We do not favor this interpretation because we have not observed the entire array of CD8 T cell responses in peripheral blood during the acute phase of infection of M3 homozygous MCMs with SIVmac239 (13, 16); several responses are detectable only later in infection. It is possible that these responses are exerting an antiviral effect that is not detectable by our immunological assays, but this seems unlikely.

We also detected accelerated variation kinetics in the ARF1_30-40_QL11 and Env_338-346_RF9 epitopes when CD8 T cell responses targeting these epitopes were present at a high frequency at or near the time of peak viral replication. It is reasonable to suggest that the rapid selection of these epitope variants could be attributed to higher viral replication at the time the CD8 T cell responses were present rather than as a function of enhanced epitope-specific CD8 T cell potency. Indeed, this observation suggests that the correlation between CD8 T cell potency and the selection of escape variants is not absolute. Thus, CD8 T cells that select for escape variants are not always the most potent; however, CD8 T cells that are potent do tend to select for sequence variants. In other words, rapid variation is not sufficient by itself to implicate a specific CD8 T cell response in protective immunity, but this approach can be used as an initial filter to identify potent CD8 T cell responses. Further studies are needed to evaluate whether CD8 T cell responses targeting different epitopes throughout infection are functionally different and, consequently, have distinct abilities to destroy virus-infected cells.

There are two important caveats to this study. First, the animal groups used were necessarily small. While we dedicated considerable resources to assembling a cohort of M3 homozygous MCMs to challenge with m3KOΔnef, we were unable to assemble a similarly sized cohort of M3 homozygous MCMs to challenge with SIVmac239Δnef. We now know that M3 homozygous animals generally exhibit high viral loads when challenged with SIVmac239 (15, 16), so we cannot formally exclude the possibility that MCMs with this genotype are predisposed to higher viral loads when infected with any SIV strain. Nonetheless, the nearly uniform complete control of SIVmac239Δnef replication in both Indian rhesus and Mauritian cynomolgus macaques (19, 20) (Fig. 2), irrespective of MHC background, makes this unlikely. In addition to the small group sizes, our m3KOΔnef controls were not MHC identical to one another. The only MCM haplotypes that do not share any MHC class I alleles with the M3 haplotype are rare (34), so animals that are fully MHC identical for these alternate haplotypes are exceptionally uncommon. Even though the animal numbers were small, we believe that the use of fully MHC-identical, M3 homozygous MCMs infected with m3KOΔnef provides the best cohort used to date to specifically study the *in vivo* antiviral efficacy of CD8 T cell responses that do not rapidly select for escape variants.

Second, we recognize that our results are confounded somewhat by the unexpectedly strong response to the cryptic ARF1_30-40_QL11 epitope. On the basis of our classification of early T cell responses that rapidly select for sequence variants, we would ablate this epitope in the pre-escaped virus if we repeated this study. We did not include ARF1_30-40_QL11 epitope variants in m3KOΔnef because we originally identified common variants that were nonsynonymous in the nine main SIV genes; we did not consider cryptic epitopes. Still, the presence of acute-phase CD8 T cells targeting ARF1_30-40_QL11 was insufficient to enable control of m3KOΔnef in M3 homozygous MCMs. It is provocative to consider that control of m3KOΔnef may have been even weaker in the absence of responses targeting ARF1_30-40_QL11.

The limited potency of acute-phase CD8 T cell responses targeting epitopes that do not rapidly accumulate variants calls into question the value of designing HIV vaccines to elicit CD8 T cell responses targeting conserved elements in the viral proteome. Conserved-element vaccines are attractive because the immunogenic sequences targeted by vaccine-specific immune responses should be present in most circulating virus populations (6, 40–42). Unfortunately, T cell responses targeting conserved or invariant epitopes are also typically subdominant in HIV infection (43). Our data suggest that these responses are not particularly effective for long-term control of viral replication, but we do not know whether CD8 T cell responses targeting epitopes that are even less variable than the ones studied here could effectively control viral replication. With the MCM model, we can repeat the strategy used to generate m3KOΔnef and serially ablate additional epitopes restricted by M3 MHC alleles. Given enough generations, we expect to elicit acute-phase CD8 T cell responses targeting epitopes that are practically invariant or potentially eliminate the entire set of CD8 T cell responses (27). Ultimately, challenging M3 homozygous MCMs with a “T cell epitope-deficient virus” could help quantify the requirement for CD8 T cell responses with reference to MHC-independent viral control.

Overall, the observations from this SIV model can be applied to our understanding of the complex interplay between host immune responses and viral evolution at the population level. As HIV continues to adapt to common HLA alleles in the population, the most potent epitopes may not be immunogenic in transmitted HIV strains, such that alternate CD8 T cell responses targeting less variable epitopes will predominate during the acute phase of infection. This might ultimately lead to less efficient viral control and accelerated disease progression, an alarming forecast with significant implications for the future of the HIV epidemic.

## Supplementary Material

Supplemental material
