# Supplementary material for: Acute-Phase CD8 T Cell Responses That Select for Escape Variants Are Needed to Control Live Attenuated Simian Immunodeficiency Virus
Source: J Virol. 2013 Aug;87(16):9353–64. doi: 10.1128/JVI.00909-13 (PMC3754066; doi:10.1128/JVI.00909-13)
Supplement: Supplemental material [file supp_87_16_9353__index.html]

Supplemental material 

# Acute-Phase CD8 T Cell Responses That Select for Escape Variants Are Needed to Control Live Attenuated Simian Immunodeficiency Virus

## Supplemental material

**Files in this Data Supplement:**

- Supplemental file 1 -

  Fig. S1 (Mapping the ARF130-40QL11 epitope.)

  Fig. S2 (*In vitro* fitness of m3KOΔnef is comparable to that of SIVmac239Δnef.)

  Fig. S3 (Immune activation in MCMs infected with m3KOΔnef or SIVmac239Δnef.)

  Fig. S4 (Genome-wide nucleotide variation at 3 weeks after infection with SIVmac239Δnef.)

  Fig. S5 (Genome-wide nucleotide variation at 3 weeks after infection with m3KOΔnef.)

  Fig. S6 (Genome-wide nucleotide variation at 12 weeks after infection with m3KOΔnef.)

  Table S1 (Average frequencies of CD38+ Ki67+ T cells in BAL fluid and blood during acute infection.)

  Table S2 (Metrics of genome-wide sequences collected for virus populations isolated after infection with m3KOΔnef or SIVmac239Δnef.)

  Table S3 (Coverage at each described epitope.)

  PDF, 456K
