# Supplementary material for: Acute-Phase CD8 T Cell Responses That Select for Escape Variants Are Needed to Control Live Attenuated Simian Immunodeficiency Virus
Source: J Virol. 2013 Aug;87(16):9353–64. doi: 10.1128/JVI.00909-13 (PMC3754066; doi:10.1128/JVI.00909-13)

Supplemental Figure Legends:

Supplemental Figure 1. Mapping the ARF1$_{30\text{-}40}$QL11 epitope. (A) Amino acid sequences in Env and ARF1 spanning the nucleotide region with detected sequence variation. (B) The frequencies of CD3+CD8+ T cells producing IFNγ and/or TNFα after incubation of the polyclonal T cell line with the indicated peptides are shown. (C) MHC class I transferent cell lines expressing the indicated MHC class I alleles were pulsed with the ARF1$_{30\text{-}40}$QL11 peptide and co-cultured with the polyclonal T cell line grown against ARF1$_{29\text{-}43}$VY15. Activation of CD8 T cells was measured by intracellular cytokine staining.

Supplemental Figure 2. The in vitro fitness of m3KOΔnef is comparable to SIVmac239Δnef. In vitro co-culture fitness assays were performed with m3KOΔnef and SIVmac239Δnef. For each assay, a barcoded SIVΔnef (BCVΔnef) was included as a reference. The barcoded virus was included at a 1:9, 1:1, or 9:1 ratio of p27 content relative to the query virus in the inoculum. The ratio of the number of copies of query virus to barcoded virus was determined at each time point relative to the ratio of the number of copies of query virus to barcoded virus present in the inoculum. Unpaired t-tests were performed at each time point.

Supplemental Figure 3. Immune activation in MCMs infected with m3KOΔnef or SIVmac239Δnef. The frequencies of CD3+CD4+ or CD3+CD8+ T cells that were also CD38+ and Ki67+ were measured in blood or cells isolated from bronchoalveolar lavage fluid (BAL) or blood on days 0, 14, and 21 after infection. The legend below the graph

indicates the MHC genotype and the infecting virus. Each line represents a different animal.

Supplemental Figure 4. Genome-wide nucleotide variation at three weeks after infection with SIVmac239Δnef. Collected sequences were aligned to SIVmac239 as described in the Materials and Methods. Variants spanning positions 1,300 to 10,200 are shown if the percent variation was >1% and greater than three times the percentage of uncertain or indel sequences. The dashed horizontal line marks variation present at 20%. The vertical dark gray box spans the 182bp sequence in *nef* that is absent in SIVmac239Δnef. Animal identification numbers are shown to the left of each graph.

Supplemental Figure 5. Genome-wide nucleotide variation at three weeks post infection with m3KOΔnef. Sequences collected were aligned to SIVmac239 as described in the Materials and Methods. Variants spanning positions 1,300 to 10,200 are shown if the percent variation was >1% and greater than three times the percentage of uncertain or indel sequences. The dashed vertical line identifies the location of $ARF1_{30-40}QL11$. The vertical dark gray box spans the 182bp sequence in *nef* that is absent in m3KOΔnef. Mutations engineered into SIVmac239 to create m3KOΔnef are shown in red. If a mutation is nonsynonymous in one reading frame, but synonymous in an alternate reading frame, then the red filled circle has a black ring. The substitution at position 6925 in m3KOΔnef is synonymous on its own, but is nonsynonymous when linked to 6923, so the black ring overlaps the red filled circle. Animal identification numbers are shown to the left of each graph.

Supplemental Figure 6. Genome-wide nucleotide variation at twelve weeks after infection with m3KOΔnef. Collected sequences were aligned to SIVmac239 as described in the Materials and Methods. Variants spanning positions 1,300 to 10,200 are shown if the percent variation was >1% and greater than three times the percentage of uncertain or indel sequences. The vertical light gray box spans a region where sequence data could not be obtained from virus populations replicating in CY0381. The vertical dark gray box spans the 182bp sequence in *nef* that is absent in m3KOΔnef. Mutations engineered into SIVmac239 to create m3KOΔnef are shown in red. If a mutation is nonsynonymous in one reading frame, but synonymous in an alternate reading frame, then the red filled circle has a black ring. The substitution at position 6925 in m3KOΔnef is synonymous on its own, but is nonsynonymous when linked to 6923, so the black ring overlaps the red filled circle. Animal identification numbers are shown to the left of each graph.

Supplemental Table 1. Average frequencies of CD38+Ki67+ T cells in BAL fluid and blood during acute infection

Group	Avg frequency of CD38+Ki67+ cells in animals infected with m3KOΔnef (n=6)	Avg frequency of CD38+Ki67+ cells in animals infected with SIVmac239Δnef (n=3)
CD3+CD4+ BAL Day 0	6.75	6.0
CD3+CD4+ BAL Day 14	36.4	15.8
CD3+CD4+ BAL Day 21	28.9	10.7
CD3+CD4+ Blood Day 0	7.94	2.29
CD3+CD4+ Blood Day 14	4.19	2.21
CD3+CD4+ Blood Day 21	10.4	1.54
CD3+CD8+ BAL Day 0	6.18	9.77
CD3+CD8+ BAL Day 14	80.0	15.7
CD3+CD8+ BAL Day 21	22.7	18.2
CD3+CD8+ Blood Day 0	6.37	1.84
CD3+CD8+ Blood Day 14	7.46	4.07
CD3+CD8+ Blood Day 21	14.7	1.93

The average frequencies of CD3+CD4+ or CD3+CD8+ T cells that are CD38+Ki67+ are shown for samples taken from the different sites at the different time points.

Supplemental Table 2. Metrics of genome-wide sequences collected for virus populations isolated after infection with m3KOΔnef or SIVmac239Δnef.

Animal & time point	# of reads	Avg. Coverage	Min. Coverage	Max. Coverage	Viral load
CY0379 3wk	60,527	1555	67	3331	36,700
CY0381 3wk	47,896	1053	28	2866	125,000
CY0384 3wk	50,878	1134	28	2964	64,500
CY0385 3wk	59,366	1301	30	3745	55,700
CY0348 3wk	42,951	1251	52	2686	9,800
CY0382 3wk	59,974	1595	36	3058	4,150
CY0383 3wk	49,072	1282	34	2513	3,750
CY0379 12wk	14,344	420	11	823	7,170
CY0381 12wk	14,484	538	11	1026	2,280
CY0384 12wk	13,322	395	14	1048	200
CY0385 12wk	12,159	381	9	719	8,680
CY0338 3wk	37,006	1062	38	2384	5,450
CY0345 3wk	36,377	1066	27	2140	5,930
CY0386 3wk	42,608	1227	44	2557	1,370

Metrics for the collected sequences are shown in the table. The total number of sequencing reads for each sample is listed. Coverage of total A, C, T, and G nucleotides at each position from 1,300 to 10,200 (excluding the deletion in *nef* from position 9507 to 9688) was quantified. The average coverage, minimum coverage, and maximum coverage are shown across these positions. The viral load (copies/ml) for each animal at the given time point is also shown.

Supplemental Table 3. Coverage at each described epitope

Animal & time point	$ARF1_{30\text{-}40}QL11$	$Env_{338\text{-}346}RF9$
CY0379 3wk	477	845
CY0381 3wk	343	939
CY0384 3wk	395	978
CY0385 3wk	428	1167
CY0332 4wk	598	--
CY0333 4wk	732	--
CY0334 4wk	307	--
CY0335 4wk	434	--
CY0336 4wk	666	--
CY0337 4wk	741	--
CY0332 12wk	137	557
CY0333 12wk	650	850
CY0334 12wk	400	450
CY0335 12wk	342	514
CY0336 12wk	499	821
CY0337 12wk	755	949
CY0379 12wk	--	294
CY0381 12wk	--	495
CY0384 12wk	--	389
CY0385 12wk	--	250

The table shows the number of total reads with complete high quality sequence data for each epitope in each population of viruses. A double dash indicates that the specific epitope was not analyzed in this manuscript for this data set.

Supplemental Figure 1

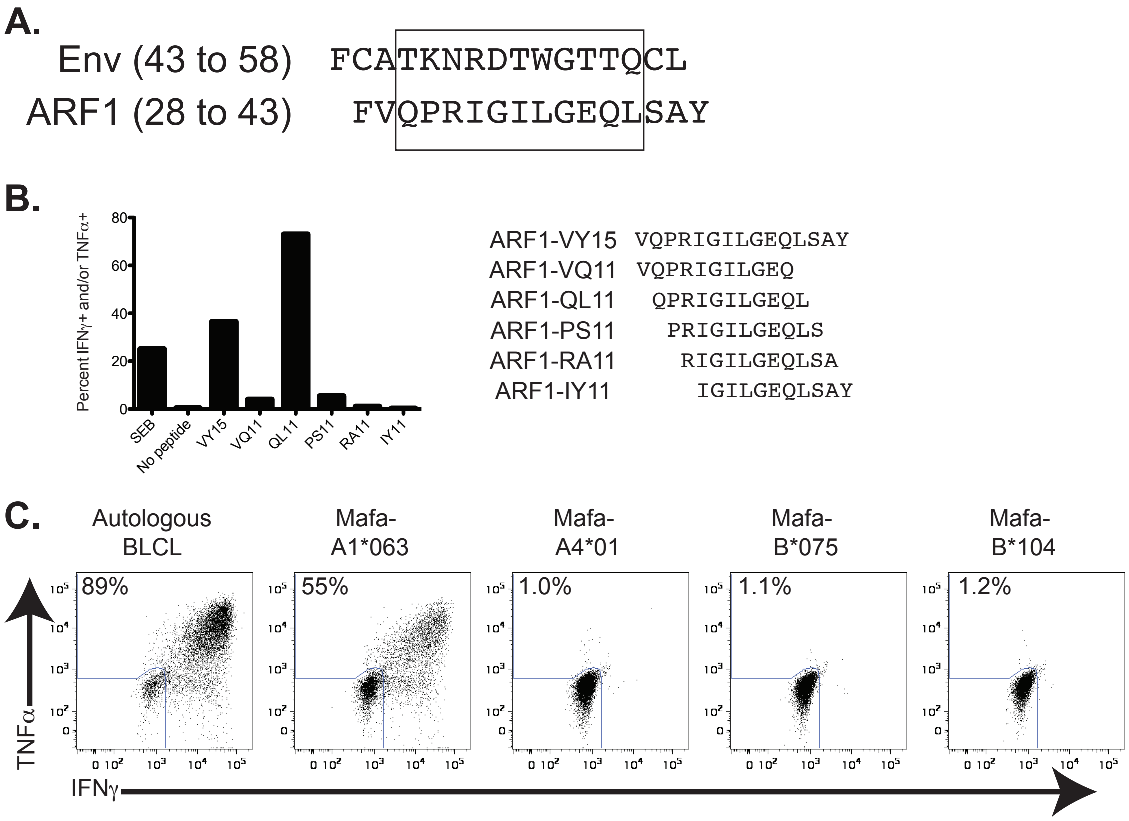

Supplemental Figure 2

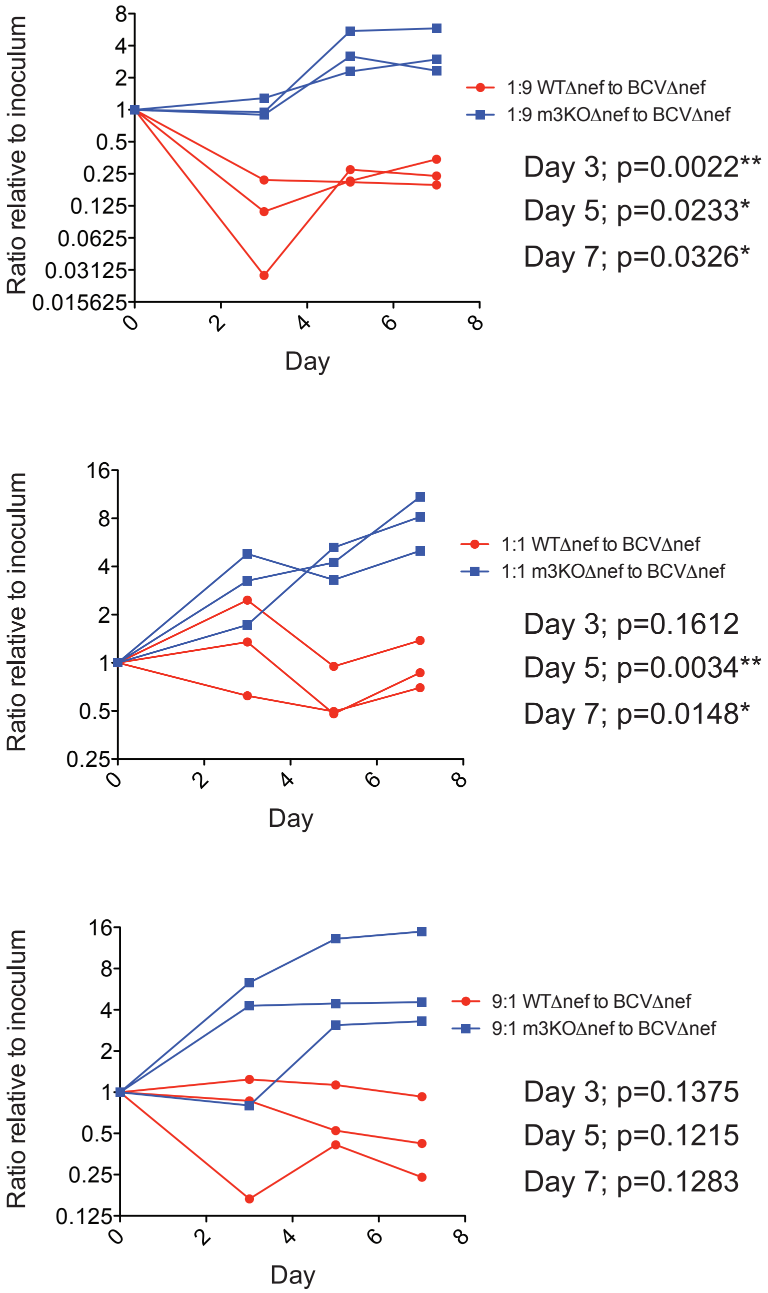

Supplemental Figure 3

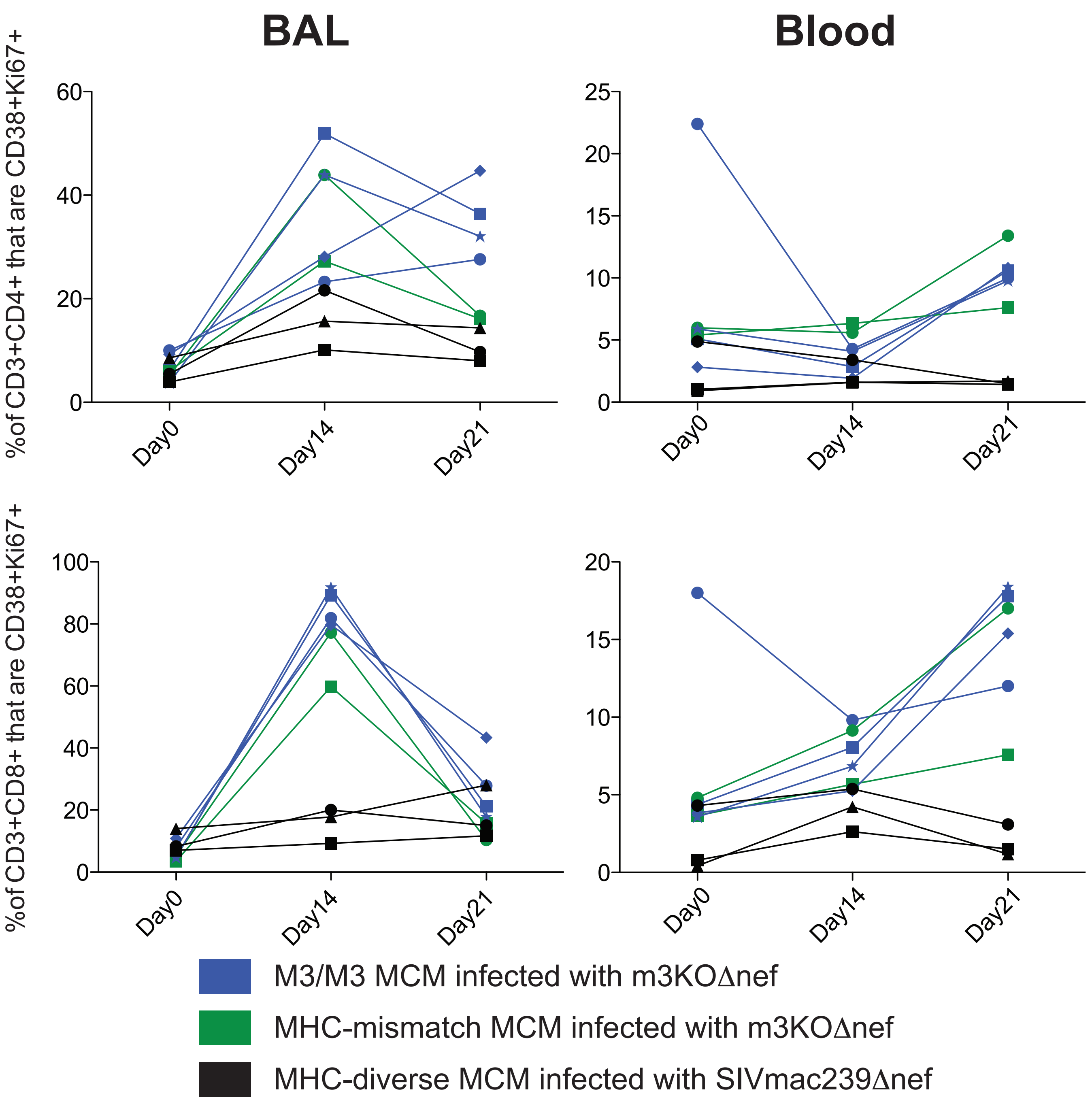

Supplemental Figure 4

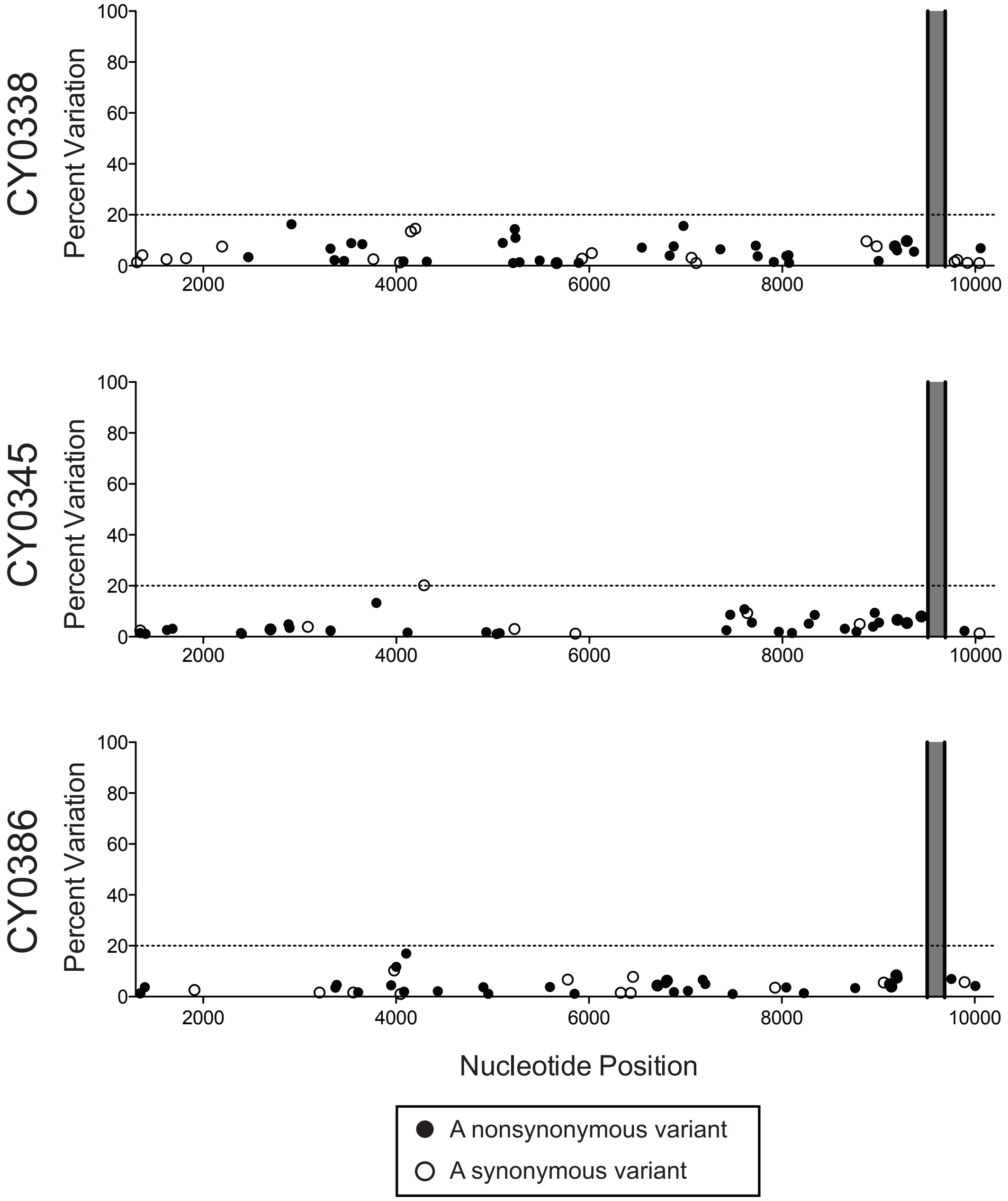

Supplemental Figure 5

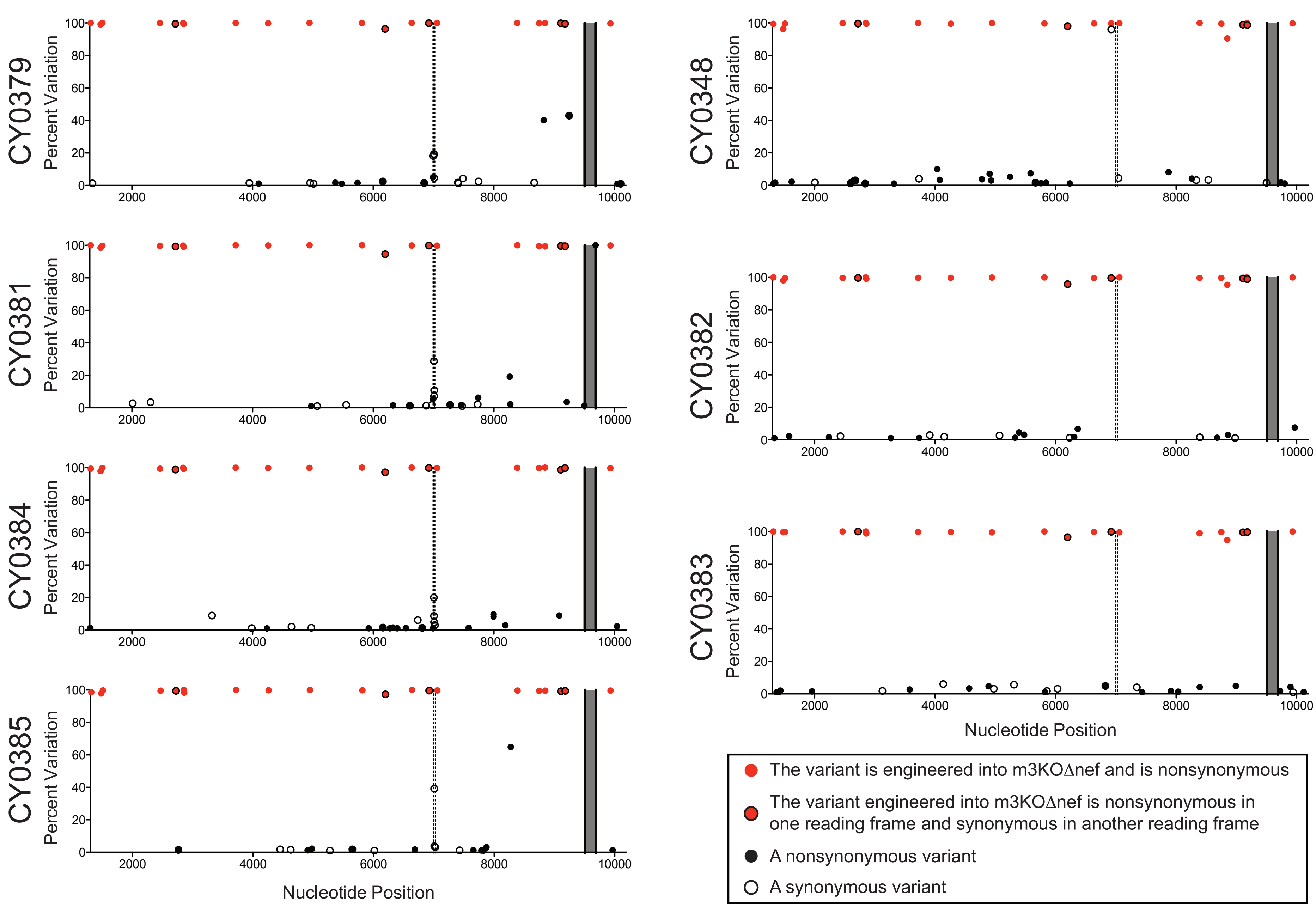

Supplemental Figure 6

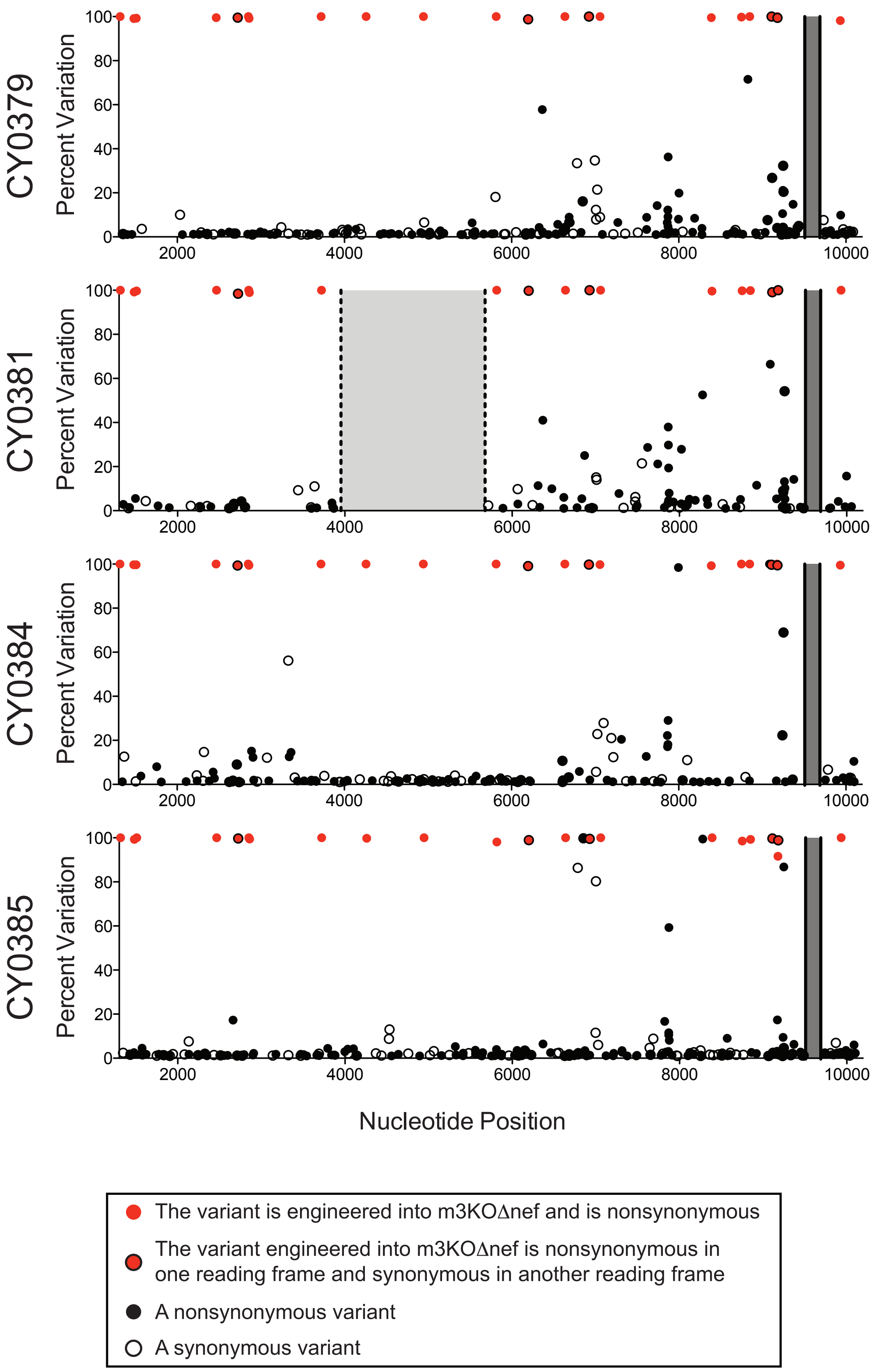

Supplement: Supplemental material [file JVI.00909-13_zjv999097986so1.pdf]
